# Cross-species protection suggests *Entamoeba histolytica* trogocytosis enables complement resistance through the transfer of negative regulators of complement activation

**DOI:** 10.1128/iai.00220-25

**Published:** 2025-07-31

**Authors:** Maura C. Ruyechan, Wesley Huang, Katherine S. Ralston

**Affiliations:** 1 Department of Microbiology and Molecular Genetics, University of California 200415, Davis, California, USA; University of California San Diego School of Medicine, La Jolla, California, USA

**Keywords:** *Entamoeba*, trogocytosis, complement, cell death

## Abstract

*Entamoeba histolytica* is a major cause of diarrheal disease. *E. histolytica* trophozoites (“amoebae”) can invade the intestine and disseminate via the bloodstream, resisting complement lysis through unknown mechanisms. Amoebae kill human cells by performing trogocytosis. After performing trogocytosis, amoebae display human proteins on their own surface and are resistant to lysis by human serum. In this study, we sought to further evaluate the mechanism by which amoebae resist complement lysis. To test if complement is responsible for lysis of amoebae, C3-depleted serum was compared to replete serum, and C3 was indeed required for lysis. Amoebae were allowed to perform trogocytosis of human cells and exposed to mouse serum. Although they had performed trogocytosis on a different species than the source of the serum, they were protected from lysis. To test if the protection from lysis by mouse serum was due to the functional interchangeability of human and mouse complement pathway proteins, human CD46 or CD55 (negative regulators of complement activation) were exogenously expressed. Amoebae that expressed human CD46 or CD55 were protected from lysis by mouse serum, indicating that display of human proteins was sufficient to inhibit mouse complement activation. Finally, amoebae were allowed to perform trogocytosis of a cell type in which the complement pathway is not conserved, and they did not become resistant to lysis. Overall, these findings are consistent with the model that trogocytosis enables amoebic acquisition and display of host proteins, including negative regulators of the complement pathway, that provide protection from complement lysis. Since other microbes can perform trogocytosis, this novel mechanism for complement resistance might apply to other infections.

## INTRODUCTION

Amoebiasis is global human diarrheal disease that is caused by the parasite *Entamoeba histolytica* (1). *E. histolytica* cysts are transmitted primarily via fecal contamination of water, and thus, the most affected countries are those with insufficient sanitation (2). Amoebiasis is estimated to cause 67,900 deaths globally per year (3, 4). Because death is a rare outcome of this infection, the global burden of amoebiasis is much higher than the number of deaths per year would suggest. In an endemic area, approximately 80% of infants are infected (5). Malnutrition and stunting are associated with childhood *E. histolytica* infections (6). In children in the Global Enteric Multicenter Study, *E. histolytica* infection was associated with the highest risk of death between enrollment and follow up (7).

In most cases, amoebiasis is asymptomatic or causes mild diarrhea. However, *E. histolytica* can be invasive, causing amoebic dysentery with bloody diarrhea. *E. histolytica* can also spread from the intestine to other organs, resulting in abscess formation in vital organs (2). The liver is most commonly affected, resulting in amoebic liver abscesses that are fatal if not treated. There is no vaccine, and therapeutic options are limited (8). There is a limited understanding of the molecular pathogenesis of disease and the determinants of the different disease outcomes.

*E. histolytica* trophozoites (“amoebae”) damage the colon during amoebic dysentery and damage vital organs during disseminated disease, thereby earning their name (*histo-:* tissue, and *-lytic:* destruction or lysis). The profound cell-killing activity of amoebae is likely to be central to tissue damage. Amoebae can kill almost any type of human cell within minutes (9, 10). Amoebic actin is required, and amoebae must be intact and viable since lysates, culture supernatants, and killed amoebae all lack cell killing activity (9–11). The previously accepted model was that amoebae secrete pore-forming “amoebapores” (12–15). However, the contact dependence of cell killing and the lack of killing activity in cell lysates and supernatants are not consistent with the presence of secreted toxins. Furthermore, transfer of amoebapores to human cells has not been experimentally demonstrated. Cysteine proteases, which are surface-localized and secreted, are key virulence factors that cleave a variety of host substrates, including mucins, antimicrobial peptides, and extracellular matrix components (16–18). Thus, cysteine proteases have a clear role in tissue damage. The surface D-galactose and N-acetyl-D-galactosamine (Gal/GalNAc) lectin is critical for attachment to substrates, including mucins and almost any type of human cell. In disseminated infections, *E. histolytica* survives in the bloodstream and, thus, must be capable of evading the host innate immune system to facilitate its passage to the organs. While it is well documented that more virulent strains of *E. histolytica* are better at evading complement lysis, it is not entirely clear what enables this property (19, 20). The heavy chain of the Gal/GalNAc lectin mimics CD59, a negative regulator of complement activation, and is, thus, one component of complement resistance (21). However, the Gal/GalNAc lectin is not sufficient to fully protect amoebae from complement lysis, as amoebae are readily lysed by human serum *in vitro* (22).

In recent years, it has become clear that amoebae kill human cells by performing cell-nibbling, known as trogocytosis (*trogo-:* nibble) (23). During trogocytosis, amoebae pinch off and internalize bites of membrane and intracellular contents from human cells, which eventually results in human cell death (23). Inhibitors and mutants that quantitatively reduce amoebic trogocytosis also reduce human cell death, suggesting that trogocytosis is the mechanism by which amoebae kill human cells (23). In addition to its role in *E. histolytica*, trogocytosis is an apparently common, but poorly understood, form of cell-to-cell interaction in eukaryotic biology, with roles in cell killing, intercellular communication, and cellular remodeling (24). When mammalian immune cells perform trogocytosis, interestingly, proteins acquired from the nibbled cell can be displayed by the cell that took bites (25–27).

Through the work of Miller et al. (22), we found that amoebae that have performed trogocytosis display human cell membrane proteins on their own surface (22). Amoebae that had performed trogocytosis were also protected from lysis by human serum (22). Human serum contains complement proteins that are a key component of the innate immune system. The complement cascade can be activated in multiple ways (i.e., via the lectin, classical, and alternative complement activation pathways), but all manners of activation converge on the cleavage of C3 into C3b which deposits on the surface of pathogens and sets further immune responses in motion (28). C3b deposition leads to further cleavage of other complement components and the formation of the membrane attack complex (MAC) that creates pores in the cell membrane leading to cell lysis.

*E. histolytica* is known to be capable of surviving in the bloodstream since the migration of amoebae from the intestine to other tissues occurs via the bloodstream, but it is not clear how *E. histolytica* resists complement activation. This is a key gap in understanding, as for a parasite that is always extracellular, perhaps nothing is more fundamental than its mechanisms of complement resistance. Although the complement resistance of amoebae involves the CD59 mimicry of the heavy chain of the Gal/GalNAc lectin (21), this alone is not sufficient to protect amoebae from lysis, as they are readily lysed upon serum exposure (22). The new finding that amoebae became complement resistant after performing trogocytosis (22) was consistent with prior work that had shown that amoebae became more resistant to complement after interacting with host cells or tissues (29–31) and that this resistance might involve proteins on the amoeba surface that can interfere with the deposition of C3b.

We hypothesized that protection from lysis by human serum was due to amoebic acquisition and display of negative regulators of the complement pathway since amoebae that had performed trogocytosis displayed human CD46 and CD59 (32). To test this, we created human cell mutants lacking individual complement regulators, but this did not sensitize amoebae to complement lysis, potentially fitting with the redundancy of negative complement regulators. Human cells typically display multiple complement regulators, and it has been shown that some, such as CD46 and CD55, both work to prevent the formation of C3b convertase on the cell surface, sometimes even degrading the same proteins. When amoebae exogenously expressed individual human complement regulators, CD46 or CD55, this was sufficient for protection from human serum lysis, showing that surface display of complement regulatory proteins can protect amoebae (32). These findings indicated that not only was trogocytosis protective, this protection was likely due to the display of human complement regulators (32).

Together, prior studies suggested a model whereby amoebae acquire and display host membrane proteins after performing trogocytosis, and the display of host proteins enables complement resistance (32). However, it was possible that performing trogocytosis generally helps amoebae resist stressors like complement activation and membrane attack complex (MAC) assembly on the amoebic surface. To further test the requirement for the display of acquired host proteins in subsequent resistance to complement lysis, amoebae were allowed to perform trogocytosis of human cells and then exposed to mouse serum. Because proteins displayed by amoebae were from a different species than the source of the serum, we did not expect amoebae that had nibbled human cells to resist lysis by mouse serum, but surprisingly, they were protected. Exogenous expression of human CD46 or CD55 protected amoebae from lysis by mouse serum, suggesting that due to the relatedness of these species, aspects of complement regulation are functionally interchangeable. To further test the requirement for display of acquired host proteins in subsequent resistance to complement lysis, amoebae were allowed to perform trogocytosis of a cell type in which the complement pathway is not conserved, and they did not become resistant to lysis by human serum. Together, these findings support that amoebic acquisition and display of host proteins, including negative regulators of the complement pathway, is required for acquired protection from complement lysis.

## RESULTS

### Killing of *E. histolytica* by human serum requires complement activation

**Fig 1:**
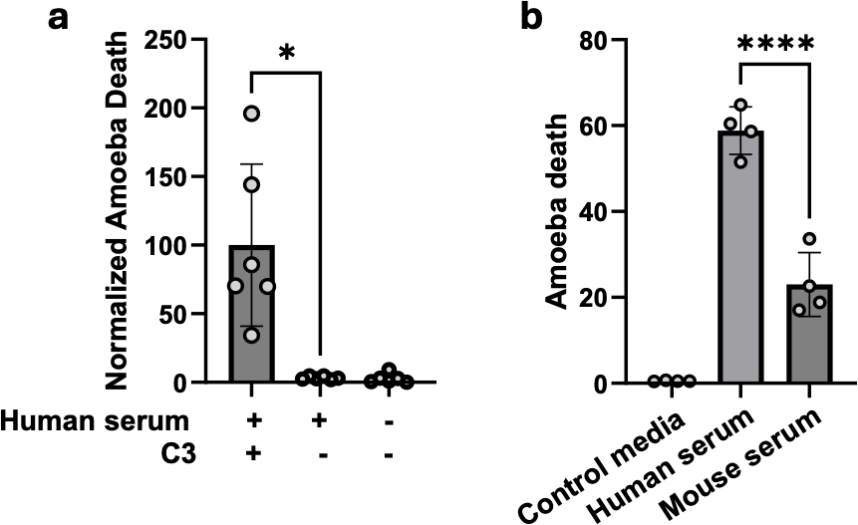
C3 is required for lysis of amoebae by human serum, and human serum lyses amoebae more efficiently than mouse serum. (**a**) Amoebae were labeled with CMFDA and exposed to complete human serum, C3-depleted human serum, or control M199s media for 30 minutes. Amoebae were stained with Live/Dead fixable violet and analyzed with imaging flow cytometry. *N* = 6 across two independent experiments. (**b**), CMFDA-labeled amoebae were exposed to human serum, mouse serum, or M199s for 30 minutes. Cells were stained with Zombie Violet dye and analyzed by imaging flow cytometry. *N* = 4 across 1 experiment. For both panels, Brown-Forsythe and Welch’s ANOVA tests with Dunnett’s T3 multiple comparisons test were used with statistical significance indicated as follows: ns, *P* > 0.05; *, *P* ≤ 0.05; **, *P* ≤ 0.01; ***, *P* ≤ 0.001; ****, *P* ≤ 0.0001.

We have previously shown that following exposure to human serum, C3b is deposited on the amoeba surface (22). Both C3b deposition and lysis of amoebae are reduced in amoebae that have performed trogocytosis, compared to amoebae that have not performed trogocytosis (22). We also showed that human serum that has been heat-inactivated does not lyse amoebae (22). Together, these findings are consistent with a requirement for complement activation for lysis of amoebae by human serum. To formally test if the complement pathway is required for lysis of amoebae by human serum, amoebae were exposed to C3-depleted human serum or complete human serum, and imaging flow cytometry was used to quantify amoeba viability (Fig. 1a; Fig. S1). C3-depleted serum led to significantly less amoeba lysis compared to complete human serum (Fig. 1a). Thus, lysis of amoebae by human serum requires complement activation.

### Amoebae are lysed by mouse serum

We extended our analysis of lysis of amoebae by serum by exposing amoebae to mouse serum. Although mice are not a natural host of *E. histolytica*, experimental infection of mice with amoebae mimics many aspects of the human infection, ranging from immune responses to the host genetic determinants of susceptibility to infection (33). To test if mouse serum led to lysis of amoebae, amoebae were exposed to mouse serum, or human serum as a control, and viability was assessed using imaging flow cytometry. Amoebae were lysed by mouse serum (Fig. S2). The complement pathway requires Ca^2+^ and Mg^2^ and serum is typically supplemented with Ca^2+^ and Mg^2+^ for *in vitro* studies of complement activation (34, 35). Mouse serum appears to require higher levels of Ca^2+^ and Mg^2+^ supplementation (36), and consistent with this, there was a trend toward higher levels of lysis with higher levels of Ca^2+^ and Mg^2+^ supplementation (Fig. S2). Mouse serum led to lower levels of lysis overall, in comparison to human serum (Fig. 1b), consistent with its known reduced complement activity (36, 37).

### Amoebae that have performed trogocytosis of human cells are protected from lysis by mouse serum

**Fig 2:**
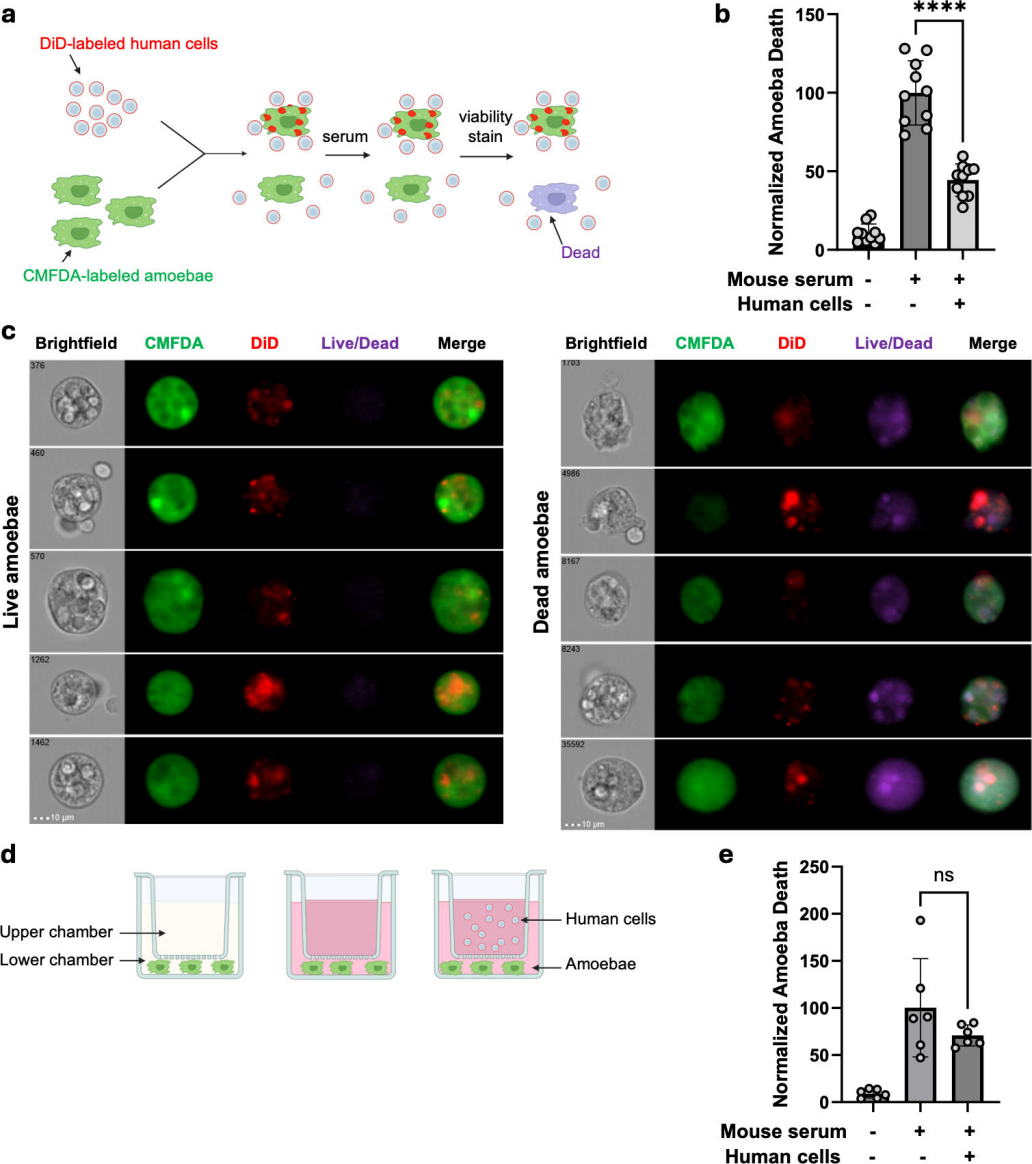
Trogocytosis of human cells protects amoebae from lysis by mouse serum. (**a and b**) As shown in the schematic, CMFDA-labeled amoebae were co-incubated with DiD-labeled human Jurkat T cells or incubated in the absence of human cells as a control. Next, cells were exposed to mouse serum or media as a control. A viability stain (Zombie Violet or Live/Dead fixable violet) was used to label dead cells, and cells were analyzed using imaging flow cytometry. *N* = 10 across 3 independent experiments. (**c**) Representative images of live and dead amoebae from the experiments in panel b, that were exposed to mouse serum following trogocytosis of human Jurkat T cells. (**d and e**) As shown in the schematic, CMFDA-labeled amoebae were incubated in the lower chamber of a transwell apparatus. Amoebae were exposed to media as a control or mouse serum. Among amoebae exposed to mouse serum, some samples also contained human Jurkat T cells in the upper chamber of the transwell apparatus. A viability stain (Zombie Violet or Live/Dead fixable violet) was used to label dead cells, and cells were analyzed using imaging flow cytometry. *N* = 5–6 across 2 independent experiments. For panels b and e, Brown-Forsythe and Welch’s ANOVA tests with Dunnett’s T3 multiple comparisons test were used with statistical significance indicated as follows: ns, *P* > 0.05; *, *P* ≤ 0.05; **, *P* ≤ 0.01; ***, *P* ≤ 0.001; ****, *P* ≤ 0.0001.

In previous studies, amoebae that had performed trogocytosis of human cells were subsequently protected from lysis by human serum (22). Protection from lysis correlated with display of human proteins on the amoeba surface (32). To further test the requirement for display of acquired host proteins in subsequent resistance to complement lysis, amoebae were allowed to perform trogocytosis of human cells and then exposed to mouse serum (Fig. 2a through c). We hypothesized that amoebae that had performed trogocytosis of human cells would not be protected from lysis by mouse serum because the host proteins displayed by amoebae would be from a different species than the source of the serum. However, amoebae that had performed trogocytosis of human cells were protected from lysis by mouse serum (Fig. 2b).

The reduced lysis of amoebae by mouse serum could be due to the presence of human cells, which might titrate mouse complement activity. To test if the presence of human cells led to a reduction in mouse serum complement activity, transwell chambers were used. Amoebae were incubated in the absence of human cells in the lower transwell chamber, or human cells were added to the upper transwell chamber (Fig. 2d and e). Trogocytosis is a contact-dependent process, therefore separating the amoebae and human cells into different chambers prevented trogocytosis while still maintaining the same ratio of cells to mouse serum. There was no significant difference in the lysis of amoebae by mouse serum, regardless of whether human cells were present (Fig. 2e). This showed that the presence of human cells does not dilute the complement activity of mouse serum.

### Amoebae that exogenously express human complement regulators are protected from lysis by mouse serum

**Fig 3:**
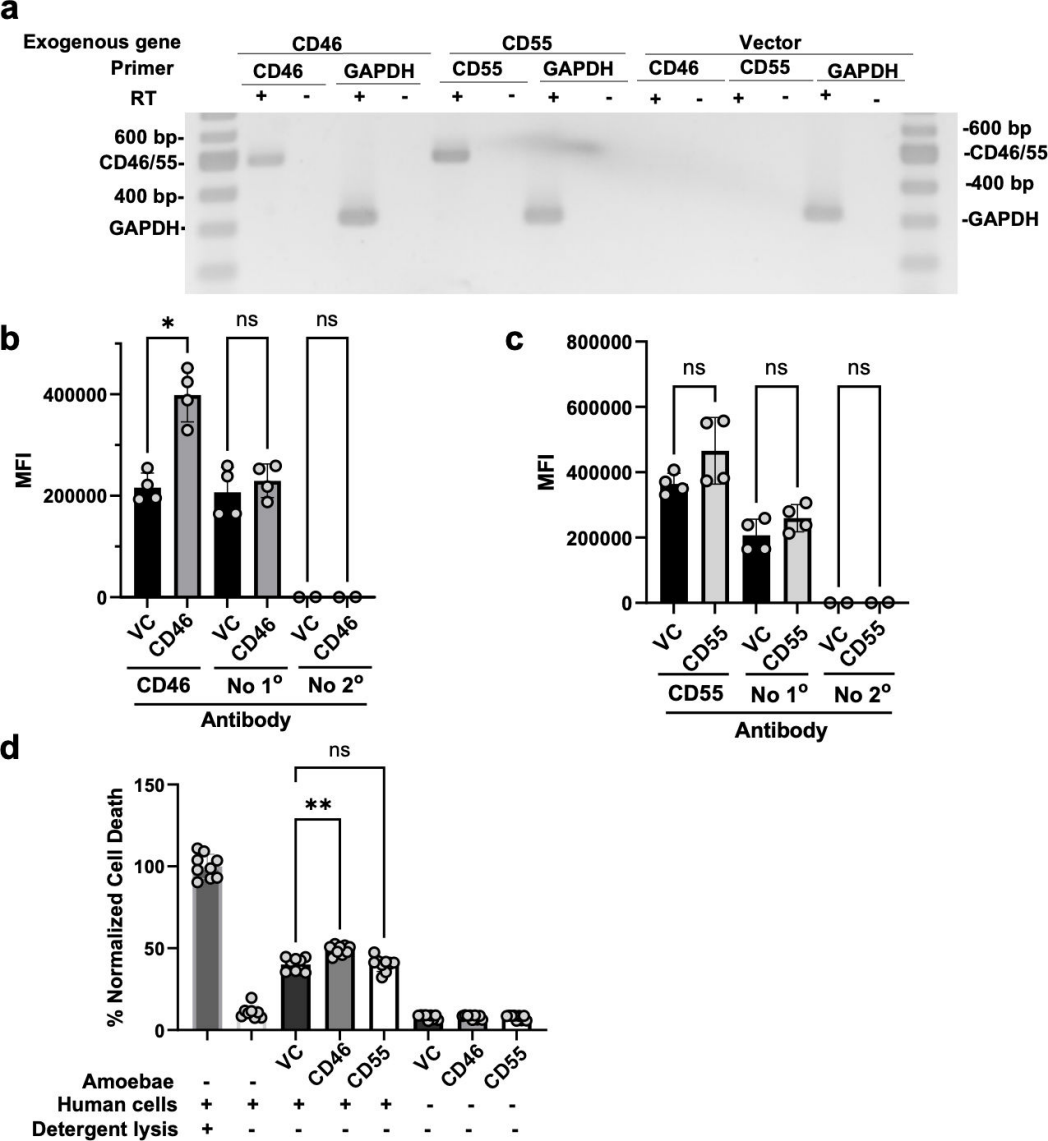
Exogenous expression of human CD46 or CD55 in *E. histolytica*. (**a**) RT-PCR analysis of amoebae stably expressing human CD46, CD55, or the corresponding plasmid vector control. Amplification of the endogenous *E. histolytica* GAPDH gene was used as a control. RT reactions were performed with (+) or without (−) reverse transcriptase, to control for the presence of residual genomic DNA. (**b and c**) Immunofluorescence assays of amoebae exogenously expressing human CD46, human CD55, or the corresponding plasmid vector control. *N* = 2-4 from 2 independent experiments. (**d**) Amoebae exogenously expressing human CD46, human CD55, or the corresponding plasmid vector control were co-incubated with human Caco-2 cells and LDH release was used as a readout for Caco-2 cell death. As controls, Caco-2 cells were incubated without amoebae, and detergent lysis was performed in order to measure LDH release in fully lysed Caco-2 samples. Amoebae were incubated in the absence of Caco-2 cells as an additional control for amoeba cell death. *N* = 9 from 3 independent experiments. For panels b-d, Brown-Forsythe and Welch’s ANOVA tests with Dunnett’s T3 multiple comparisons test with statistical significance indicated as follows: ns, *P* > 0.05; *, *P* ≤ 0.05; **, *P* ≤ 0.01; ***, *P* ≤ 0.001; ****, *P* ≤ 0.0001.

**Fig 4:**
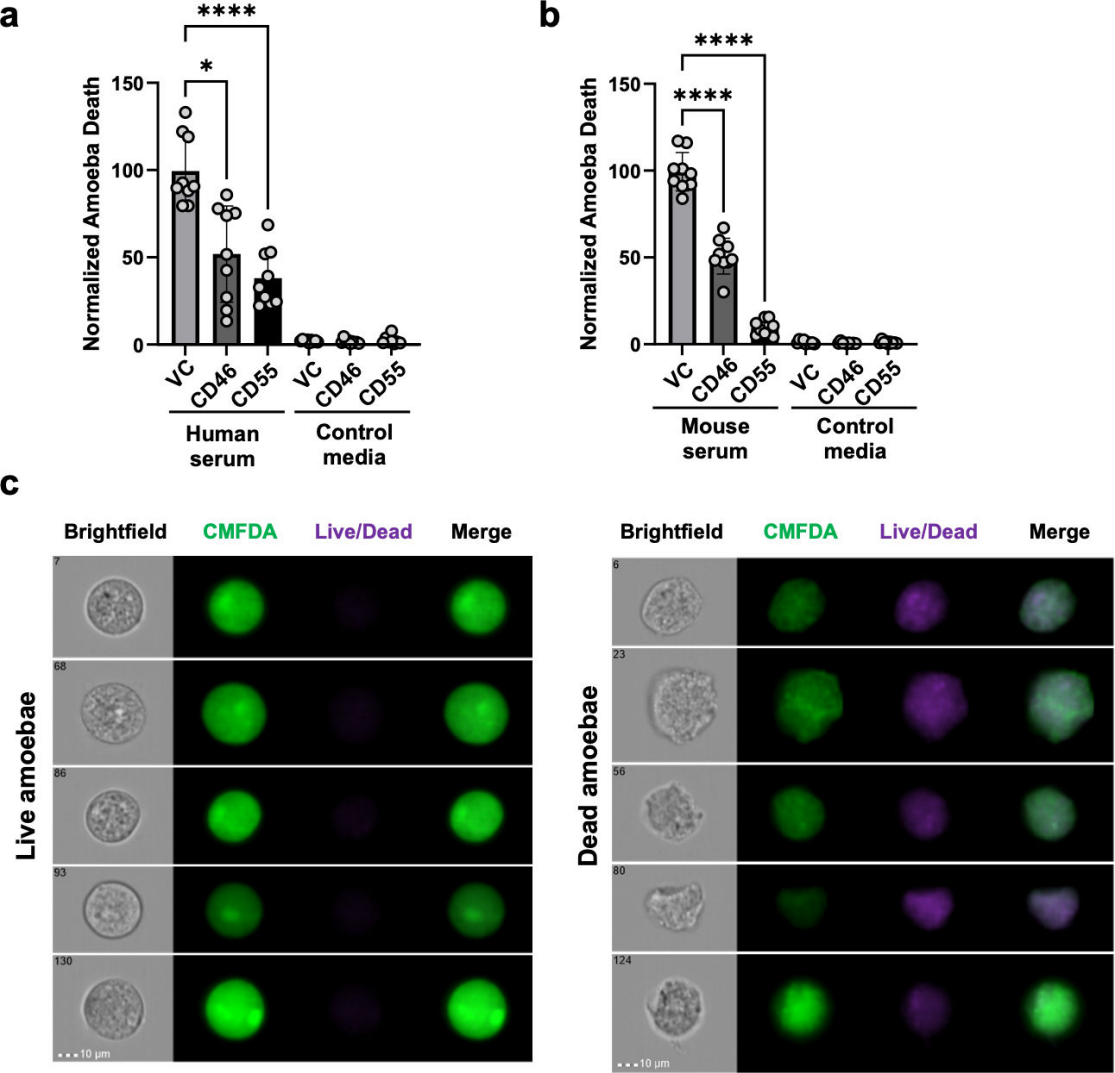
Exogenous expression of human CD46 or CD55 protects amoebae from lysis by both human and mouse serum. (**a and b**) CMFDA-labeled amoebae exogenously expressing human CD46, CD55, or the corresponding plasmid vector control were exposed to human or mouse serum for 30 mins. Amoebae were stained with Live/Dead fixable violet and analyzed with imaging flow cytometry. *N* = 8–9 across 3 independent experiments for panel a and *N* = 9 across three independent experiments for panel b. (**c**) Representative images of live and dead amoebae from the experiments in panel b. For panels a and b, Brown-Forsythe and Welch’s ANOVA tests with Dunnett’s T3 multiple comparisons test with statistical significance indicated as follows: ns, *P* > 0.05; *, *P* ≤ 0.05; **, *P* ≤ 0.01; ***, *P* ≤ 0.001; ****, *P* ≤ 0.0001.

We initially hypothesized that amoebae that had performed trogocytosis of human cells would not be protected from lysis by mouse serum because the host proteins displayed by amoebae would be from a different species than the serum. However, because the complement pathway is conserved, it is possible that the displayed human proteins were similar enough to mouse complement proteins to inhibit complement activation. We previously showed that amoebae that exogenously express the negative regulators of complement, CD46 or CD55, are protected from lysis by human serum. Therefore, to ask if human and mouse complement proteins could be functionally interchangeable in this context, we next asked if amoebae exogenously expressing human CD46 or CD55 (Fig. 3a through c) would be protected from mouse serum. This experiment built upon our previous finding that amoebae displayed CD46 after performing trogocytosis of human cells and that exogenous expression of human CD46 or CD55 in amoebae was sufficient to protect amoebae from lysis by human serum (32). The difference here is that we asked if exogeneous expression of the same human complement regulators could protect amoebae from lysis by mouse serum. While the expression level of CD46 or CD55 was low (Fig. 3b and c), it is notable that the endogenous expression level of these proteins in human Jurkat T cells is also low (Fig. S3). There was a small increase in the cell-killing activity of CD46-expressing amoebae compared to vector control transfectants (Fig. 3d). As expected, the exogenous expression of CD46 or CD55 protected amoebae from lysis by human serum (Fig. 4a). Exogenous expression of CD46 or CD55 also protected amoebae from lysis by mouse serum (Fig. 4b and c). This suggests that human and mouse CD46 and CD55 are functionally interchangeable in protecting amoebae from lysis by human or mouse serum.

### Amoebae that have performed trogocytosis of insect cells are not protected from lysis by human serum

**Fig 5:**
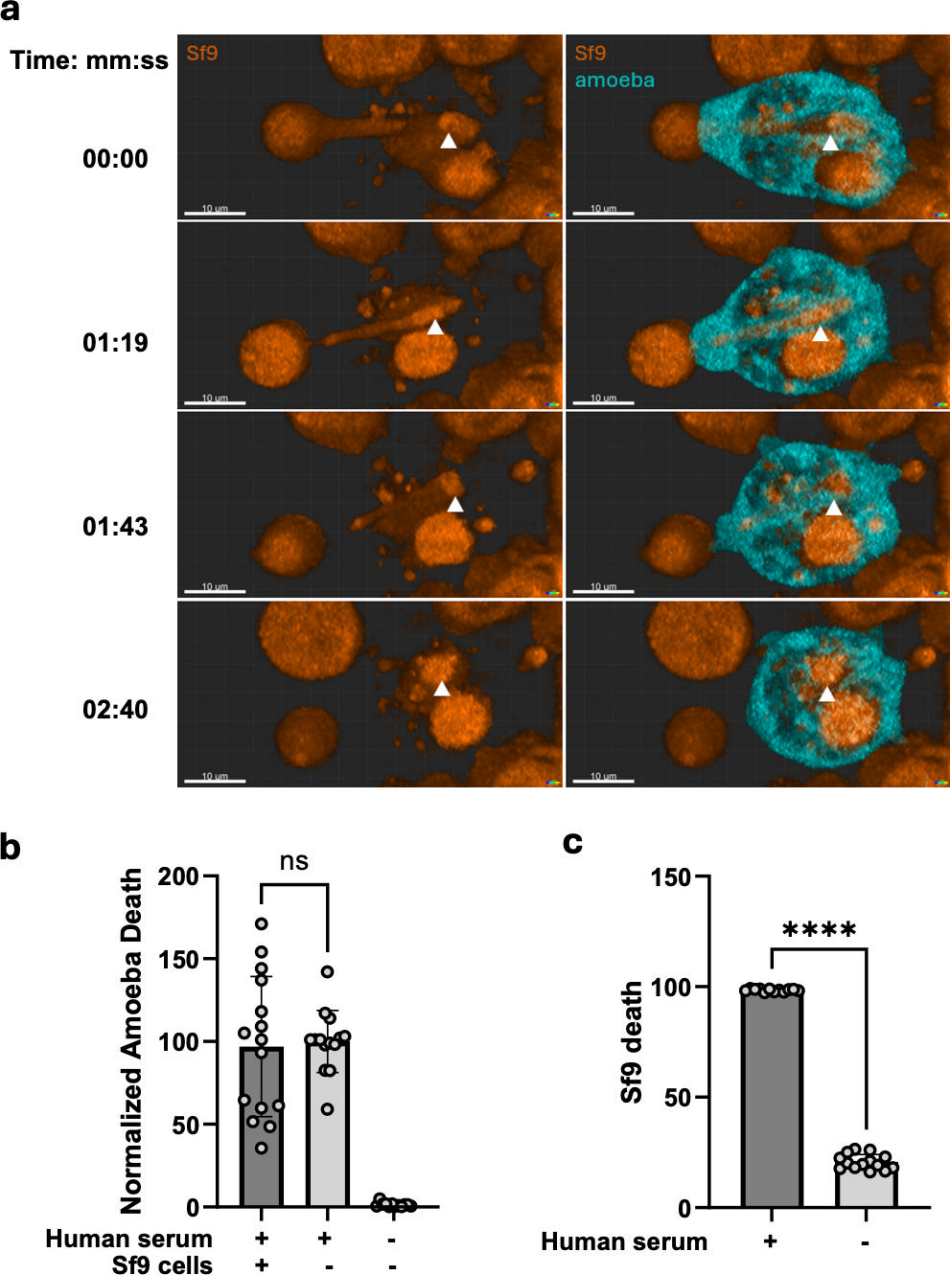
Trogocytosis of Sf9 cells does not protect amoebae from lysis by human serum. (**a**) CMFDA-labeled amoebae were co-incubated with CMTPX-labeled Sf9 cells, and imaged live on a Zeiss LSM 980 with Airyscan2. Airyscan functionality was used at the maximum speed to capture rapid *z* stacks. Images were acquired as a top-down view of entire 3D projection processed and displayed through Blend Rendering Mode in Imaris and Zeiss Zen. The white arrowhead follows a bite of the Sf9 cell as it is nibbled by the amoeba. See Video S1 for the video from which the images were extracted. (**b**) CMFDA-labeled amoebae were co-incubated with CMTPX-labeled Sf9 cells for 1 hour, or incubated in the absence of Sf9 cells, and then exposed to human serum for thirty minutes. Cells were stained with Live/Dead fixable violet and analyzed with imaging flow cytometry. (**c**) CMPTX-labeled Sf9 cells were incubated in the absence of amoebae and then exposed to human serum. *N* = 12–15 across five independent experiments. For panel b, Brown-Forsythe and Welch’s ANOVA tests with Dunnett’s T3 multiple comparisons test were used. For panel c, an unpaired *t*-test with Welch’s correction was used. Statistical significance is indicated as follows: ns, *P* > 0.05; *, *P* ≤ 0.05; **, *P* ≤ 0.01; ***, *P* ≤ 0.001; ****, *P* ≤ 0.0001.

To further test the requirement for display of acquired host proteins in subsequent resistance to complement lysis, amoebae were allowed to perform trogocytosis of a cell type in which the complement pathway is not conserved. Consistent with the previously described ability of amoebae to ingest and kill many different cell types (38), amoebae performed trogocytosis of Sf9 insect cells (Fig. 5a; Videos S1 to S3). Amoebae that performed trogocytosis of Sf9 cells were not subsequently protected from lysis by human serum (Fig. 5b; Fig. S4). As expected, since the complement pathway is not conserved in Sf9 cells, when Sf9 cells were exposed to human serum as a control, they were efficiently lysed (Fig. 5c). Together, these findings support that amoebic acquisition and display of host proteins, which include functional negative regulators of the complement pathway, is required for subsequent resistance to lysis.

## DISCUSSION

Prior work suggested that post-trogocytosis display of host cell membrane proteins by amoebae leads to complement resistance (22, 32). To further test this model, in the current studies, amoebae were allowed to perform trogocytosis of human cells and then exposed to mouse serum, and surprisingly, they were protected from complement lysis. We found that amoebae exogenously expressing human complement regulators resisted mouse complement lysis, suggesting that the display of human proteins can functionally inhibit mouse complement activation. Amoebae that performed trogocytosis of Sf9 cells, in which the complement pathway is not conserved, did not become resistant to lysis by human serum. Together, these findings support that amoebic acquisition and display of host proteins, including negative regulators of the complement pathway, is required for acquired protection from complement lysis.

In the current studies, C3-depleted human serum did not lyse amoebae (Fig. 1). This is consistent with prior work that showed that heat inactivation, which inhibits complement proteins, renders human serum unable to lyse amoebae (22). This finding is also consistent with prior studies that showed that amoebae that were killed after serum exposure were intensely decorated with C3b (22). Together, these findings demonstrate that the lysis of amoebae by human serum is due to complement activation.

Here, to further test the requirement for the display of acquired host proteins in subsequent resistance to complement lysis, amoebae were allowed to perform trogocytosis of human cells and then exposed to mouse serum. We did not expect amoebae that had nibbled human cells to resist lysis by mouse serum, but surprisingly, they were protected. Exogenous expression of human CD46 or CD55 also protected amoebae from lysis by mouse serum suggesting that due to the relatedness of these species, aspects of complement regulation are functionally interchangeable. Indeed, the mouse and human complement systems are very similar in their mechanism of action.

The negative regulators of complement activation are similar in amino acid sequence in mice and humans (Table 1). These regulators are critical to prevent the inadvertent activation of the complement system and the lysis of self cells. In both mice and humans, surface-localized negative regulators of complement include CD46 (Membrane Cofactor Protein) and CD55 (Decay Accelerating Factor). CD46 degrades C3b and C4b, while CD55 degrades C3b; thus, both prevent the formation of the MAC (39, 40). In mice, CD46 is predominantly found in the testes and retina, and a separate protein, Crry (complement receptor 1-related protein/gene y), is more widely expressed and performs similar functions to both CD46 and CD55 (41, 42). Mouse and human CD46 share ~47% identity and ~64% similarity, with mouse Crry having ~29% identity to human CD46 and ~44% similarity. Mouse and human CD55 protein isoforms share ~47% identity and ~62% similarity. Mouse and human C3 share ~77% identity and ~88% similarity, and mouse and human C4b share ~76% identity and ~87% similarity. That the human complement regulatory proteins could provide such protection to amoebae while not having high levels of identity with mouse proteins likely indicates that there is sufficient similarity between these two species’ complement regulatory proteins and their substrates to enable human CD46 or CD55 to inhibit mouse complement activation.

**TABLE 1:** Comparison of human and mouse complement and complement regulatory proteins^*a*^

Human protein accession number	Mouse protein accession number	% Identity	% Similarity
CD46 NP_002380.3	CD46 NP_034908.1	~47	~64
CD46 NP_002380.3	Crry NP_001341989.1	~29	~44
CD55 NP_001108224.1	CD55 NP_001391888.1	~47	~62
C3 NP_000055.2	C3 NP_033908.2	~77	~88
C4b NP_001002029.3	C4b NP_033910.2	~76	~87

^*a*^ Human and mouse complement and complement regulatory proteins were aligned, and the percentage identity and similarity between the human and mouse proteins are indicated. Additionally, mouse Crry and human CD46 were compared. Mouse Crry and human CD46 have a more similar distribution in expression and may function more similar to one another than mouse CD46 and human CD46 (41, 42).

To our knowledge, this is first the time that lysis of *E. histolytica* by mouse serum has been evaluated. Mouse serum led to lower levels of amoebic lysis in comparison to human serum (Fig. 1b). These data were consistent with findings from an analysis of the complement activity of wild-type mouse and human serum performed by Latuczek et al. (2014) wherein mouse serum had 10 times lower complement activity compared to human serum when tested via a hemolytic CH_50_ assay (37). The reasons for these differences in lytic activity are not currently known but could potentially be due to differences in the how the different complement pathways function together in each species, as seen in the exposure of dextran-coated SPIO nanoworms to human and mouse serum (43).

Similar to the previous studies in which a transwell was used to separate amoebae and human cells, our findings in Fig. 2e are consistent with a lack of a role for secreted factors in amoebic complement resistance. In our prior work, transwells were used to separate amoebae from human cells, and then amoebae were exposed to human serum (22). Amoebae only became resistant to lysis by human serum if they were incubated on the same side of the transwell as human cells (22). This suggested that secreted factors from human cells, such as proteins or extracellular vesicles, did not have a role in amoebic resistance to complement lysis. Moreover, these findings further affirmed that trogocytosis was likely to be required for complement resistance since trogocytosis requires direct cell-cell contact. Here, we extended these findings by showing that amoebae that are separated from human cells by a transwell do not resist lysis by mouse serum. This further affirms that secreted factors from human cells are not relevant to amoebic resistance to serum and extends this finding to mouse serum.

Consistent with our previous work (32), amoebae exogenously expressing human CD46 or CD55 were protected from lysis by human serum (Fig. 4a). In our previous work (32), amoebae were stably transfected with plasmids to drive the expression of either CD46 or CD55 and then exposed to increased concentrations of the selective antibiotic (G418) in order to induce overexpression of the exogenous protein. This is a common approach in the *E. histolytica* research community for overexpression (44–47). In the present work, we replaced the promoter in the overexpression construct, with a promoter that drives higher levels of gene expression (48). This allows for overexpression, without the need to raise the level of the selective antibiotic. This is a preferable approach since raising the selective antibiotic likely imposes deleterious effects on amoebae. The consistency between our prior findings with overexpression of CD46 and CD55 using raised G418 (32) and the current findings using a stronger promoter (Fig. 4a) demonstrate that the stronger promoter is just as effective. We suggest that this approach is preferable to increased drug selection and is a new tool that is available for the *E. histolytica* research community. Finally, amoebae exogenously expressing CD46 were noted to have slightly higher cell-killing activity compared to vector control transfectants. The difference was statistically significant, but because the difference was very small, it is not clear that this difference would be meaningful in the context of an infection.

We found that amoebae perform trogocytosis of Sf9 cells. This fits with the known broadness of amoebic cell-killing activity. Sf9 cells are likely to have glycosylated surface proteins that the amoebic Gal/GalNAc lectin could bind, as they have glycosylation pathways which produce n-linked glycoproteins with high mannose glycans and glycans with trimanosyl cores (49, 50). While amoebae were able to attach to Sf9 cells and perform trogocytosis, they did not become resistant to lysis by human serum. The insect immune system does use TEPs or thioester-containing proteins. These function similar to the mammalian complement system, as complement proteins are a subfamily of thioester-containing proteins, while those in insects are specifically known as insect TEPs (51). Insect TEPs are also noted to be much more similar to α−2M subfamily of thioester-containing proteins than to complement proteins (51). It is as of yet unknown if there are any insect TEP homologs for complement proteins (51). Our findings indicate that any TEPs present on the Sf9 cell surface are not sufficiently similar to human complement proteins, as there was near total death of Sf9 cells upon exposure to human serum (Fig. 5c).

There are many remaining questions about trogocytosis and immune evasion by *E. histolytica*. While it is evident that host surface proteins are trafficked to the amoebic surface, the pathway by which they are trafficked has not yet been discerned. It is also not known if the display of human proteins impacts other aspects of host-parasite interactions, beyond complement evasion. Overall, our findings indicate that trogocytosis by *E. histolytica* enables a novel mechanism for complement resistance, through the display of negative complement regulators, thereby exploiting the immune system’s own regulatory mechanisms. Since other microbes perform trogocytosis (52, 53), there is the potential for protein display to apply to the pathogenesis of other infections. Further studies of protein trafficking during amoebic trogocytosis may also improve understanding of protein trafficking during eukaryotic trogocytosis in general.

## MATERIALS AND METHODS

### Cell culture

The HM-1:IMSS strain of *E. histolytica* trophozoites from ATCC was cultured as described previously (22, 32, 54). Briefly, amoebae were cultured at 35°C in TYI-S-33 medium supplemented with 80 U/mL penicillin, 80 µg/mL streptomycin (Gibco), 2.3% Diamond vitamin Tween 80 solution (40×; Sigma-Aldrich, or prepared according to (55)), and 15% heat-inactivated adult bovine serum (Gemini Bio-Products) (55, 56). Amoebae were maintained in either unvented T25 tissue culture flasks or glass tissue culture tubes and passaged when they reached 80% confluence. Amoebae were harvested at 80%–90% confluence for experiments (22, 32).

Human Jurkat T cells from ATCC (clone E6-1) were cultured as described (22, 32). Briefly, Jurkat cells were cultured at 37°C and 5% CO2 in RPMI 1640 medium (Gibco; RPMI 1640 with L-glutamine and without phenol red) supplemented with 10% heat-inactivated fetal bovine serum (Gibco or Summa Life Sciences), 100 µg/mL streptomycin, 100 U/mL penicillin, and 10 mM HEPES. Jurkat T cells were maintained in T25 vented tissue culture flasks and expanded in T75 vented tissue culture flasks. Jurkat cells were passaged when numbers reached between 5 × 10^5^ and 2 × 10^6^ cells/mL and were harvested at the same cell concentrations for experiments (22, 32).

Human Caco-2 cells were cultured at 5% CO_2_ at 37°C in 1× MEM (Gibco) supplemented with 20% fetal bovine serum (Summa), 1% non-essential amino acids (Gibco), 1% sodium pyruvate (Gibco), 100 µg/mL streptomycin, and 100 U/mL penicillin. Caco-2 cells were maintained in T75 vented tissue culture flasks (Corning) and were passaged every 3–4 days when at 90% confluence and were harvested at the same cell concentration for experiments.

Sf9 cells from ATCC were cultured at 28°C in Grace’s Insect medium (ThermoFisher) supplemented with 10% heat-inactivated fetal bovine serum (Gibco or Summa Life Sciences). Sf9 cells were maintained in T25 vented tissue culture flasks and expanded in T75 vented tissue culture flasks. Sf9 cells were passaged every 2–3 days at ratios of 1:2 or 1:3.

For the trogocytosis, serum lysis, and live cell imaging experiments (described below), cells were harvested and resuspended in M199 medium (Gibco medium M199 with Earle’s salts, L-glutamine, and 2.2 g/L sodium bicarbonate, without phenol red) supplemented with 5.7 mM L-cysteine, 25 mM HEPES, and 0.5% bovine serum albumin. The supplemented M199 medium is referred to as M199s (22, 32).

### DNA constructs

To make plasmids for exogeneous expression of human CD55 or CD46 in *E. histolytica*, the *E. histolytica* expression plasmid pEhEx (57) was modified to replace the 5′ and 3′ sequences of the cysteine synthase (CS) gene with the 5′ and 3′ sequences of actin in order to increase the expression level of the exogeneous gene. pEhEx-RLUC, containing a Renilla luciferase (RLUC) insert, was used. pEhEx-RLUC was created by PCR amplifying the RLUC sequence from the pcDNA3 RLUC plasmid (58) (Table 2), and the pEhEx plasmid backbone, using Phusion High Fidelity polymerase (ThermoFisher). Gibson assembly with a Gibson Assembly Ultra kit (VWR) was used to ligate the sequences, resulting in the pEhEx-RLUC plasmid.

**TABLE 2:** Primers used in these studies^*a*^

Purpose	Primer	Orientation	Sequence
PCR amplification of RLUC insert for creation of pEhEx-RLUC	Renilla FWD Renilla REV	Forward Reverse	5′ AAACATTAACAGATCTTCATGACTTCGAAAGTTTATGA 3′ 5′ GAAGAGTTCAACTCGAGTGGTTATTGTTCATTTTTGAGAA 3′
PCR amplification of pEhEx backbone	pEhEx (renilla) FWD pEhEx (renilla) REV	Forward Reverse	5′ TTCTCAAAAATGAACAATAACCACTCGAGTTGAGAAAG 3′ 5′ TCATAAACTTTCGAAGTCATGAAGATCTGTTAATGTGTTT 3′
Sequencing pEhEx-RLUC	CS 5′ UTR primer CS 3′ UTR primer	Forward Reverse	5′ TCAGTCTTACCACGTCATAAAGT 3′ 5′ TGCAAGAAGATGTTACAAAGCA 3′
Amplification of Actin 5′ sequence from gDNA	RLUC pActin 107290 IF RLUC pActin 107290 IR	Forward Reverse	5′ actttcgaagtcatCTTAAGTTAATGATAATTTATTTTAGTTTTTTTGTTTAAATATCAG 3′ 5′ cttgatgggaattcCCCGGGTTTATTTTTGGAACAATAAAAATGTAAATTTAACTAAAAT 3′
PCR amplification of pEhEx-RLUC vector excluding CS 5′ sequence	RLUC 5' P/UTR BBF RLUC 5' P/UTR BBR	Forward Reverse	5′ CTTAAGatgacttcgaaagtttatgatccagaac 3′ 5′ gaattcccatcaagcttgatatcgaattcc 3′
PCR amplification of Actin 3' sequence from gDNA	Actin 3UTR IR Actin 3UTR IF	Forward Reverse	5′ aaaatgaacaataaCTTAAGTTTAGTTTAATTTCTTATTTTATTTTCAATATCTTTTTAA 3′ 5′ caaaagctgggtacGCTAGCGGATTATCTAAATTACCACCTGAAC 3′
PCR amplification of pEhEx-RLUC backbone, excluding CS 3' sequence	RLUC 3UTR BBF2 RLUC 3UTR BBR2	Forward Reverse	5′ TTAAACTAAACTTAAGttattgttcatttttgagaactcgctcaacgaac 3′ 5′ GATAATCCGCTAGCgtacccagcttttgttccctttagtgag 3′
Cloning human CD46 into pEhActEx	gc_Actin_hCD46_F2 gc_Actin_hCD46_R2	Forward Reverse	5′ AAAACTAAAATAAATTATCATTAACTTAAGATGGAGCCTCCCGGCCGCCG 3′ 5′ AATAAAATAAGAAATTAAACTAAACTTAAGTCAGCCTCTCTGCTCTGCTG 3′
Cloning human CD55 into pEhActEx	gc_ActinpEhEx_hCD55_F1 gc_ActinpEhEx_hCD55_R1	Forward Reverse	5′ AAAACTAAAATAAATTATCATTAACTTAAGATGACCGTCGCGCGGCCGAG 3′ 5′ AATAAAATAAGAAATTAAACTAAACTTAAGCTAAGTCAGCAAGCCCATGG 3′
Sequencing Vector Control (pEhActEx)	gc_ActinpEhEx_seq_1F gc_ActinpEhEx_seq_1R	Forward Reverse	5′ TGCTGCCAACACTCTTTTCA 3′ 5′ TGGGCTGCAGGAATTCGATA 3′
Sequencing pEhActEx-CD46	APCD46_seq_2F APCD46 seq 2R	Forward Reverse	5′ ACCGCTCCAAATTGCTACTG 3′ 5′ CTCCTCTTGCCACCCATACT 3′
Sequencing pEhActEx-CD55	APCD55 seq 2F APCD55 seq 2R	Forward Reverse	5′ CCAGCACCACCACAAATTGA 3′ 5′ GCCACTCCACTCTCCTTCAT 3′
RT-PCR detection of CD46 and CD55	GAPDH Forward GAPDH Reverse CD46 RT-PCR F CD46 RT-PCR R CD55 RT-PCR F CD55 RT-PCR R	Forward Reverse Forward Reverse Forward Reverse	5′ CGTCCACAGACAATTCGAAGGAAC 3′ 5′ AAGGCAGTTGGTTGTGCATGA 3′ 5′ TCCCTGCAAATGGGACTTAC 3′ 5′ GGGGGATCCCAAGTACTGTT 3′ 5′ ATGAGTGCCGTCCAGGTTAC 3′ 5′ CTGAACTGTTGGTGGGACCT 3′

^*a*^ Primers that were used for molecular cloning of plasmid constructs, Sanger sequencing of plasmid constructs, or RT-PCR analysis of transfected amoebae.

To replace the CS 5′ sequence with the actin 5′ sequence, the backbone of pEhEx-RLUC was amplified using KAPA high-fidelity polymerase (Roche), while excluding the CS 5′ sequence. A similar process was followed to replace the CS 3′ sequence with the actin 3′ sequence. The actin sequences (upstream and downstream of EHI_107290) were amplified from genomic DNA. Gibson assembly with a Gibson Assembly Ultra kit (VWR) was used to ligate the sequences, creating pEhActEx-RLUC.

Next, the coding sequences for human CD46 and CD55 were PCR amplified from pEhEx-CD46 or pEhEx-CD55 (32). These sequences were originally amplified from cDNA and, thus, lack introns. The CD46 sequence corresponds to the mRNA transcript variant d (GenBank accession number NM_153826). The CD55 sequence corresponds to the mRNA transcript variant 1 (GenBank accession number NM_000574). pEhActEx was digested with AflII to remove RLUC and the CD46 and CD55 PCR products were each cloned into the backbone using a Gibson Assembly Ultra kit (VWR). The vector control plasmid was created by self-ligating pEhActEx following removal of RLUC by AflII digestion, by using T4 DNA Ligase (NEB). Restriction digest analysis and Sanger sequencing were used to confirm all plasmid sequences. All primer sequences can be found in Table 2.

### Transfection

Amoebae were transfected as described previously (32, 54), by using 20 µg of pEhActEx, pEhActEx-CD46, or pEhActEx-CD55 using Attractene transfection reagent (Qiagen). Stably transfected amoebae were first selected at 3 µg/mL Geneticin (Thermo Fisher Scientific) and then maintained at 6 µg/mL Geneticin (32, 54).

### Gene expression analysis

RNA was extracted using the Direct-zol MiniPrep Plus kit (Zymo Research). RT-PCR was performed as previously described using the primers in Table 2 (31). Briefly, RNA was extracted and reverse transcription was used to create cDNA that was then used as a template for PCR. In order to detect genomic DNA contamination of RNA samples, a control reaction was performed for each sample, in which the reverse transcriptase enzyme was omitted. Primers to amplify the endogenous *E. histolytica* GAPDH gene were used for positive control reactions for each sample (32). RNA was extracted once per cell line, and two independent technical replicates of RT-PCR were performed.

### Immunofluorescence assays

Amoebae stably transfected with pEhActEx, pEhActEx-CD46, or pEhActEx-CD55 were washed twice with M199s medium, and fixed with 4% paraformaldehyde for 30 minutes at room temperature. Human Jurkat T cells were washed and fixed in the same manner. Samples were blocked in 1× PBS containing 0.1% Tween-20 (Sigma Aldrich), 20% normal goat serum (Jackson ImmunoResearch Laboratories), and 5% BSA for approximately 24 hours at 4°C. Samples were labeled with monoclonal antibodies directed to human CD46 (clone C-10; Santa Cruz Biotechnology), human CD55 (clone NaM16-4D2; Santa Cruz Biotechnology), or human CD3 (clone HIT3a; Santa Cruz Biotechnology), and incubated overnight at 4°C on a rocker. Samples were washed with 1×-PBS 0.1% Tween-20 and then labeled with Cy5 AffiniPure goat anti-mouse secondary antibody (Jackson Immunoresearch Laboratories, Inc.), and incubated for 3 hours at room temperature on a rocker. Samples were washed with 1×-PBS 0.1% Tween-20, resuspended in 1× PBS and analyzed on an Amnis ImageStreamX Mark II. Samples were gated by size and between 5,000 and 10,000 events were collected per sample. Data for amoebae are from 2 to 4 replicates and 2 independent experiments. Data from Jurkat T cells consist of one sample for each condition from one experiment.

### Serum lysis assays

Amoebae were washed in M199s and labeled with CMFDA at 186 ng/mL for 10 minutes at 35°C. Jurkat cells were washed in M199s and labeled with DiD at 21 µg/mL in M199s for 5 minutes at 37°C and 10 minutes at 4°C. CMFDA-labeled amoebae and DiD-labeled Jurkat cells were washed in M199s, combined at a 1:40 ratio in M199s, and co-incubated for 1 h at 35°C. As a control, amoebae were incubated under the same conditions in the absence of Jurkat cells. Next, cells were pelleted at 400 × *g* for 8 minutes and were resuspended in 100% C57BL/6 mouse serum (pooled C57BL/6 complement serum; Innovative Research Inc.), 100% human serum (pooled human complement serum; Innovative Research), or M199s depending on the sample and experiment. Mouse serum was supplemented with 75 mM CaCl_2_ and 250 mM MgCl_2_ (36). Human serum was supplemented with 150 µM CaCl_2_ and 500 µM MgCl_2_ (32). Cells were incubated in serum or M199s for 30 minutes at 35°C. Cells were washed and resuspended in M199s and incubated with either Live/Dead fixable violet (Invitrogen) or Zombie Violet (BioLegend) that was prepared as per manufacturer’s instructions at 2 µL/mL for 30 minutes on ice. Samples were fixed with 4% paraformaldehyde for 30 minutes at room temperature. Fixed samples were washed once in 1× PBS, resuspended in 1× PBS and analyzed on an Amnis ImageStreamX Mark II. 10,000 events per sample were collected for samples without Jurkat cells and 100,000 events per sample were collected for samples with Jurkat cells present. Because complement activity in human serum is variable, experiments with at least 3,000 in-focus images of amoebae per replicate (Fig. S1d), and average amoeba death in the positive control of at least 10% (Fig. S1e) were assessed.

For experiments with Sf9 cells, amoebae were labeled as described above. Sf9 cells were washed in M199s and labeled with CellTracker red CMTPX (Invitrogen) at 590.5 ng/µL for 10 minutes at 28°C. CMFDA-labeled amoebae and CMTPX-labeled Sf9 cells were washed in M199s, combined at a 1:40 ratio in M199s and co-incubated for 1 h at 28°C. As a control, amoebae were incubated under the same conditions in the absence of Sf9 cells. Next, cells were pelleted at 200 × *g* for 5 minutes and were resuspended in 100% human serum (pooled human complement serum; Innovative Research) or M199s. Human serum was supplemented as described above. Cells were incubated for 30 minutes at 35°C in either human serum or M199s. Cells were washed, stained with Live/Dead fixable violet, fixed, washed, and analyzed as described above. 10,000 events per sample were collected for samples without Sf9 cells, and 20,000 events per sample were collected for samples with Sf9 cells present. The same cutoffs as above (3,000 in-focus amoebae per replicate and >10% amoeba death in the positive control) were used in the data analysis.

For experiments with amoebae exogenously expressing human proteins, amoebae were labeled with CMFDA as described above. CMFDA-labeled amoebae were washed in M199s and exposed to human or mouse serum supplemented as described above for 30 minutes at 35°C. Cells were washed, stained with Live/Dead fixable violet, fixed, washed, and analyzed as described above. 10,000 events were collected per sample. The same cutoffs as above (3,000 in-focus amoebae per replicate and >10% amoeba death in the positive control) were used in the data analysis.

For experiments with C3-depleted serum, amoebae were labeled with CMFDA, as described above. CMFDA-labeled amoebae were washed in M199s and then resuspended in either M199s, 100% human serum (pooled human complement serum; Innovative Research), or 100% C3-depleted human serum (C3-depleted serum, human; Millipore Sigma) for 30 minutes. Serum was supplemented as described above. Cells were washed, stained with Live/Dead fixable violet, fixed, washed, and analyzed as described above. 10,000 events were collected per sample. The same cutoffs as above (3,000 in-focus amoebae per replicate and >10% amoeba death in the positive control) were used in the data analysis.

### Transwell assays

Amoebae were washed in M199s and labeled with CMFDA at 93 ng/mL in M199s for 10 minutes at 35°C. Jurkat cells were washed in M199s and labeled with DiD at 21 µg/mL in M199s for 5 minutes at 37°C and 10 minutes at 4°C. Mouse serum was supplemented with 75 mM CaCl_2_ and 250 mM MgCl_2_. CMFDA-labeled amoebae were washed in M199s and resuspended in either mouse serum or M199s. Jurkat cells were resuspended in mouse serum. Amoebae for each condition were placed in the bottom wells of a 12-well transwell plate with 3 µm pores (Corning) and the upper chamber was filled with either M199s, mouse serum, or Jurkat cells resuspended in mouse serum (at a ratio of 1:40 amoebae:human cells). Cells were incubated in a Gaspak bag (BD Biosciences) for 30 minutes at 35°C. Transwells were removed, and M199s was added to each well. The plate was then placed back in the Gaspak bag and placed on ice for 5 minutes to detach amoebae for harvesting. Amoebae were washed and resuspended in M199s media and incubated with Live/Dead fixable violet dead cell stain (Invitrogen) or Zombie Violet (Biolegend) that was prepared as per manufacturer’s instructions using 4 µL/mL for 30 minutes on ice. Samples were fixed with 4% paraformaldehyde for 30 minutes at room temperature. Fixed samples were washed once in 1× PBS, resuspended in 1× PBS, and analyzed on an Amnis ImageStreamX Mark II. 10,000 events were collected per sample of amoebae exposed to M199s, and 20,000 events were collected for samples consisting of amoebae exposed to mouse serum with or without Jurkat cells. Because complement activity in mouse serum is variable, experiments with at least 3,000 in-focus images of amoebae per replicate (Fig. S1d), and average amoeba death in the positive control of at least 10% (Fig. S1e) were assessed.

### Live cell imaging

Amoebae were washed in M199s and labeled with CMFDA at 349 ng/mL in M199s for 15 minutes at 35°C. Sf9 cells were washed in M199s and labeled with CMTPX at 591 ng/mL in M199s for 10 minutes at 28°C. CMFDA-labeled amoebae and CMTPX-labeled Sf9 cells were washed in M199s and co-incubated in an Attofluor Cell Chamber (Thermo Fisher Scientific) at 35°C without CO_2_ regulation. Cells were initially located using 10-20× objectives and imaged using 63× objectives, on a Zeiss LSM 980 with Airyscan2. Airyscan functionality was used at the maximum speed, capturing cells in the *z*-plane with an acquisition speed ranging from 80 to 140 ms. Images were processed with Zeiss Zen imaging software. Processing included a denoise step with a standard length of 1.5–2 to reduce background. The resulting files were converted to Imaris software format, and the final video and image processing was performed in Imaris (Oxford Instruments).

### LDH release cell death assays

Caco-2 cells were plated on Costar black-walled clear-bottomed 96-well plates at 1 × 10^4^ cells per well 2 days prior to performing LDH release cell death assays. On the day of the LDH release assay, Caco-2 cells were washed twice with M199s. Amoebae were washed twice in M199s and added to wells at 3 × 10^3^ cells/well. M199s was added to control wells. Plates were incubated for 3 hours at 35°C in a Pak Anaero (Mitsubishi Gas Chemical) container. To enable a measurement of 100% cell lysis, some control wells containing only Caco-2 cells were lysed. For lysis of control wells, 4 µL of lysis solution (Promega CytoTox-ONE Homogeneous Membrane Integrity Assay) was added, and plates were incubated at room temperature in a Pak Anaero (Mitsubishi Gas Chemical) container for 10 minutes. The LDH release assay was then carried out as per manufacturer instructions (Promega CytoTox-ONE Homogeneous Membrane Integrity Assay). Three experiments were carried out on different days, and data were normalized such that control lysed wells were considered to represent 100% lysis of Caco-2 cells.

### Bioinformatic analyses

Assessment of the relatedness of human and mouse complement proteins and complement regulators was performed. NCBI BLAST was used to identify human and mouse homologs of each protein. The percent identity and similarity between these proteins were assessed after aligning the proteins using Snapgene.

### Statistical analyses

Analyses were performed in Prism (GraphPad). Brown-Forsythe and Welch’s ANOVA tests with Dunnett’s T3 multiple comparisons test were used for analyses for Fig. 1, 2b, e, 3b through d, 4a, b, 5b; Fig. S4, as these assays’ experimental groups did not have equal variances. An unpaired *t*-test with Welch’s correction was used for analyses for Fig. 5c, as only two groups were present. *P* values are indicated on each figure: ns, *P* > 0.05; *, *P* ≤ 0.05; **, *P* ≤ 0.01; ***, *P* ≤ 0.001; ****, *P* ≤ 0.0001.

## SUPPLEMENTAL MATERIAL

Fig. S1 iai.00220-25-s0001.tiff Gating strategy for serum lysis experiments.

Fig. S2 iai.00220-25-s0002.tiff Optimization of mouse serum supplementation with CaCl_2_ and MgCl_2_.

Fig. S3 iai.00220-25-s0003.tiff Immunofluorescence assays of human Jurkat T cells.

Fig. S4 iai.00220-25-s0004.tiff Non-normalized data from Figure 5B.

Supplemental material iai.00220-25-s0005.docx Supplemental figure and video legends.

Video S1 iai.00220-25-s0006.mp4 Live-cell imaging of amoebic trogocytosis of Sf9 cells, example 1.

Video S2 iai.00220-25-s0007.mp4 Live-cell imaging of amoebic trogocytosis of Sf9 cells, example 2.

Video S3 iai.00220-25-s0008.mp4 Live-cell imaging of amoebic trogocytosis of Sf9 cells, single channel view of example 2.

ASM does not own the copyrights to Supplemental Material that may be linked to, or accessed through, an article. The authors have granted ASM a non-exclusive, world-wide license to publish the Supplemental Material files. Please contact the corresponding author directly for reuse.
