# Supplementary figures and images for: Cross-species protection suggests Entamoeba histolytica trogocytosis enables complement resistance through the transfer of negative regulators of complement activation
Source: Infect Immun. 2025 Jul 31;93(9):e00220-25. doi: 10.1128/iai.00220-25 (PMC12418739; doi:10.1128/iai.00220-25)

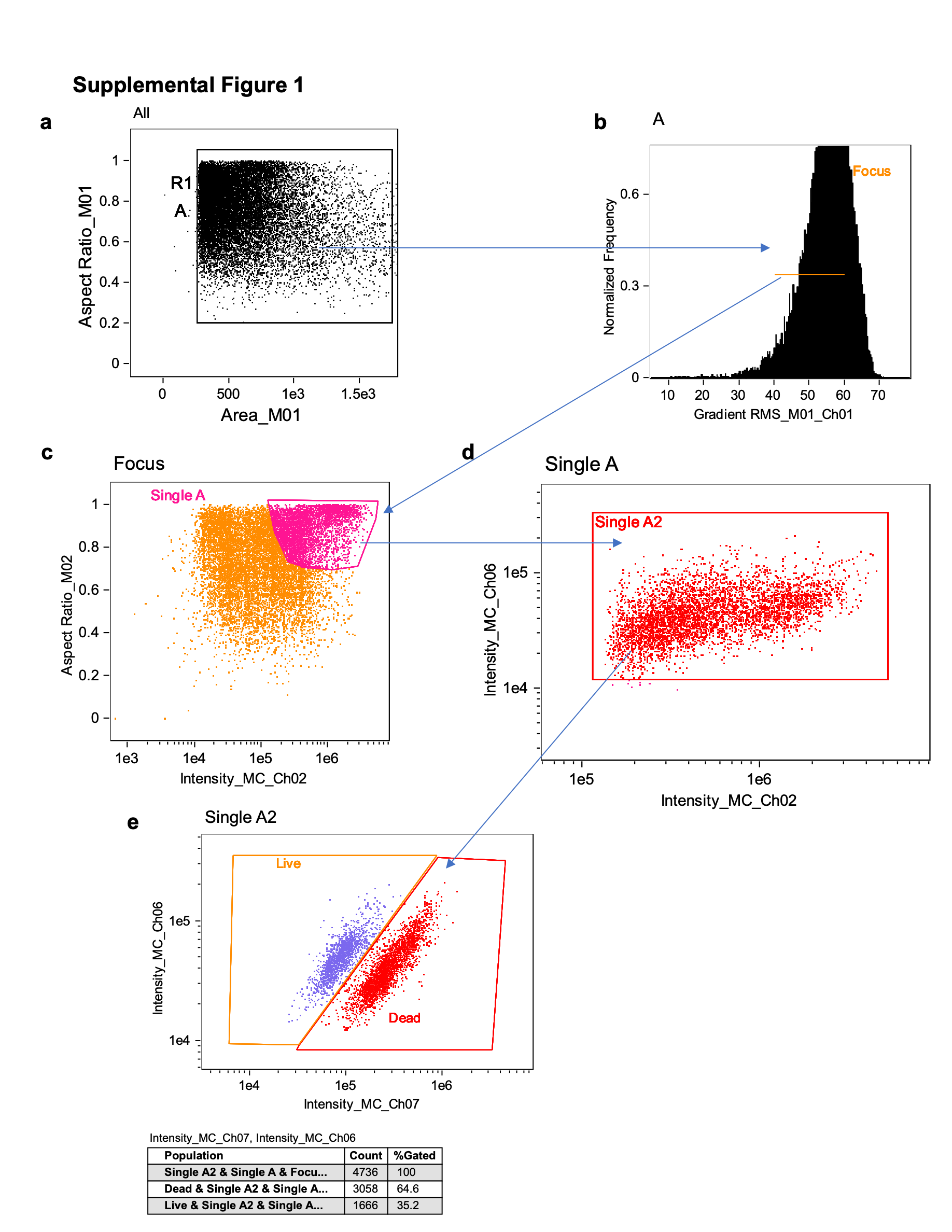

Supplement: Fig. S1 — Gating strategy for serum lysis experiments. [file iai.00220-25-s0001.tiff]

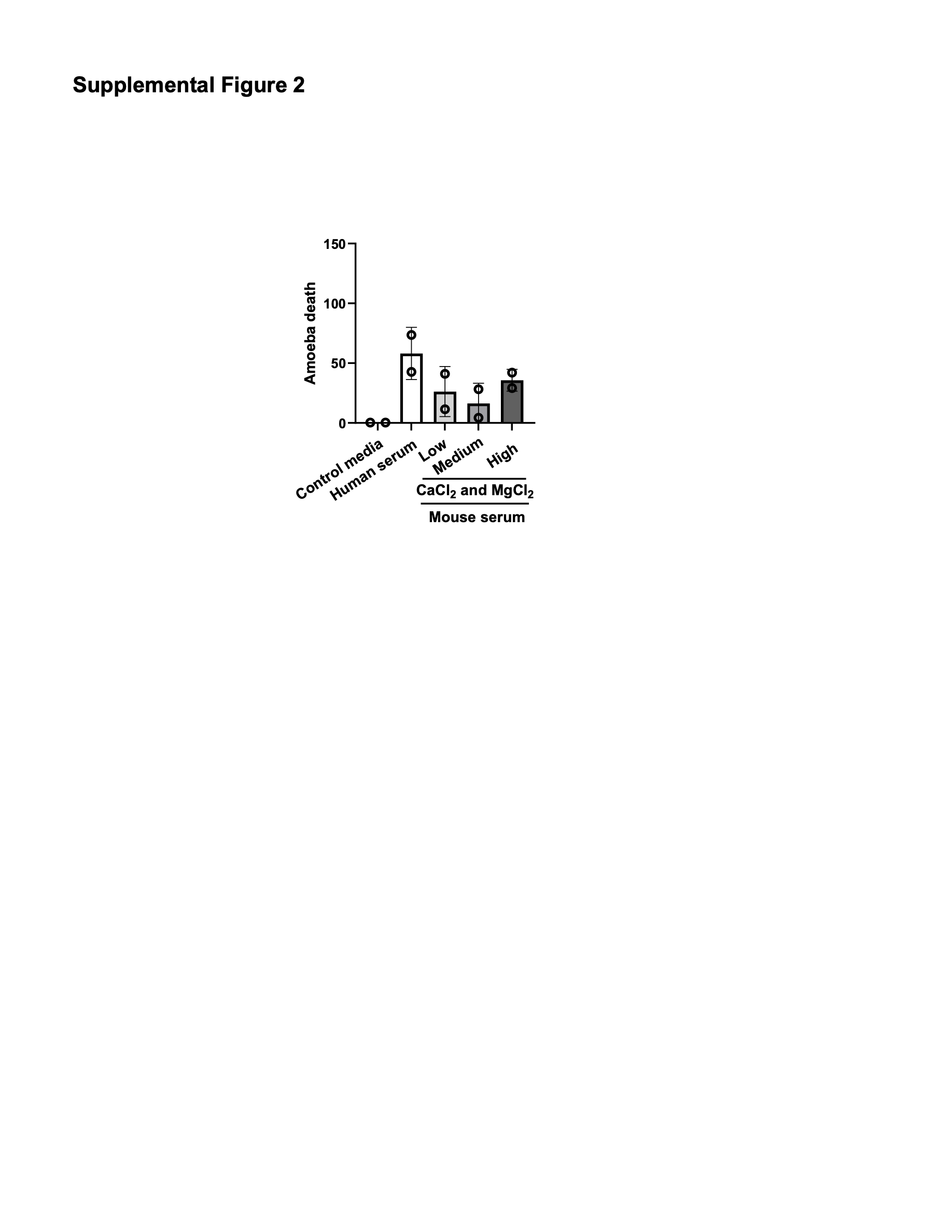

Supplement: Fig. S2 — Optimization of mouse serum supplementation with CaCl2 and MgCl2. [file iai.00220-25-s0002.tiff]

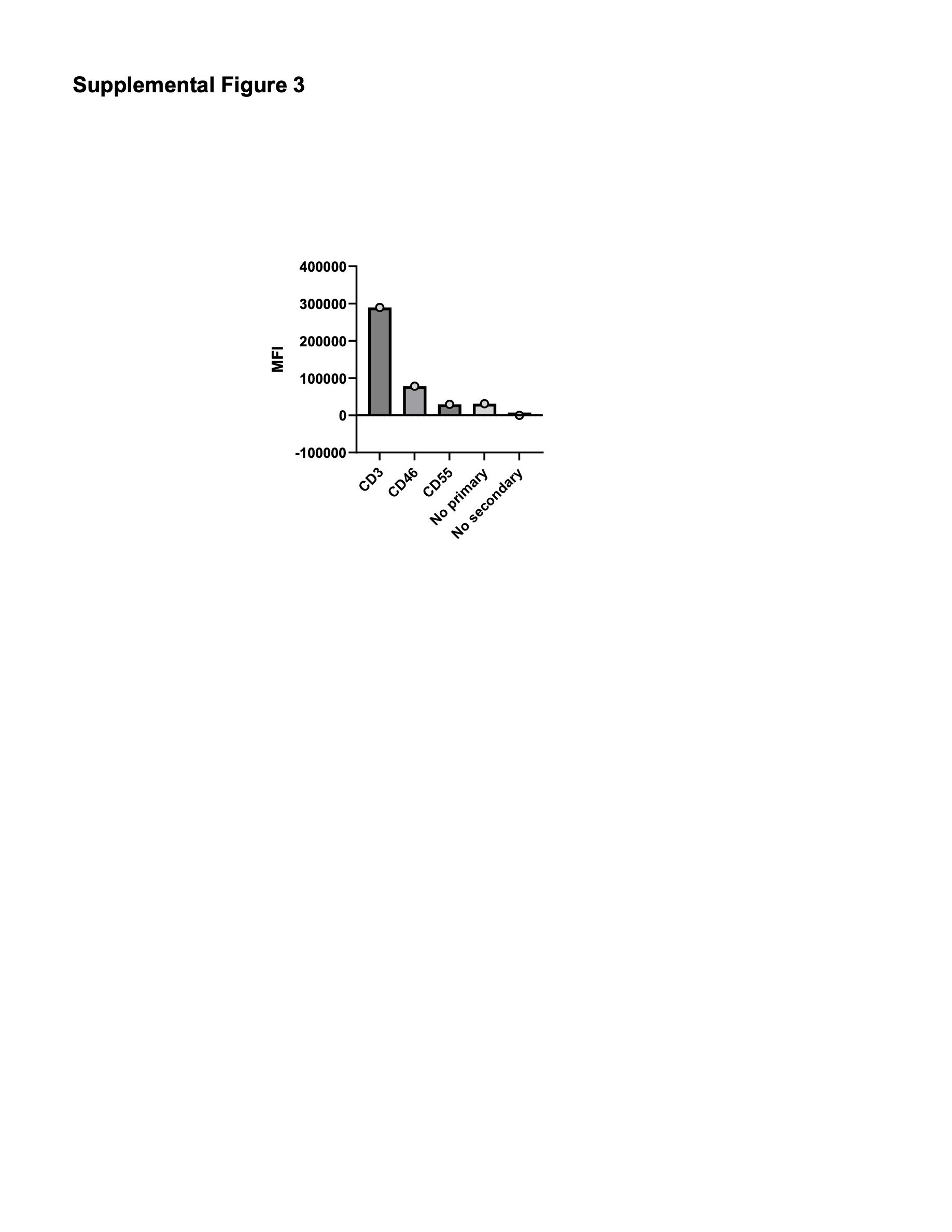

Supplement: Fig. S3 — Immunofluorescence assays of human Jurkat T cells. [file iai.00220-25-s0003.tiff]

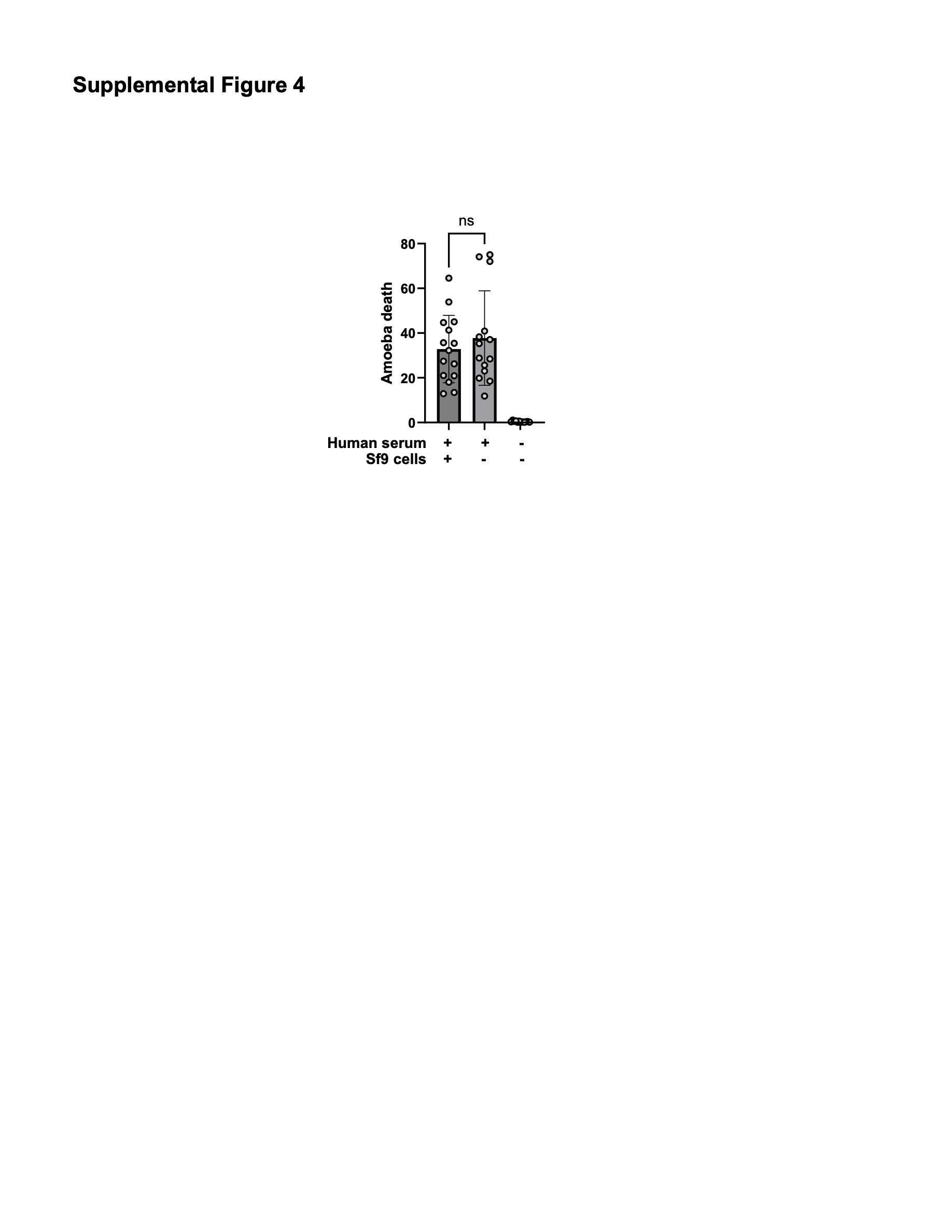

Supplement: Fig. S4 — Non-normalized data from Figure 5B. [file iai.00220-25-s0004.tiff]
