# Supplementary material for: Cross-species protection suggests Entamoeba histolytica trogocytosis enables complement resistance through the transfer of negative regulators of complement activation
Source: Infect Immun. 2025 Jul 31;93(9):e00220-25. doi: 10.1128/iai.00220-25 (PMC12418739; doi:10.1128/iai.00220-25)
Supplement: Supplemental material — Supplemental figure and video legends. [file iai.00220-25-s0005.docx]

**SUPPLEMENTARY MATERIAL**

**SUPPLEMENTAL FIGURE LEGENDS**

**Supplemental Figure 1. Gating strategy for serum lysis experiments.** **a,** All collected images were first gated using aspect ratio and area of the masked brightfield image to remove debris. **b,** Next, images were gated using the gradient RMS of the masked brightfield image to identify images that were in focus. **c,** Images were further gated by aspect ratio and intensity of the channel 2 image (corresponding to CMFDA), and images with single amoebae were gated. **d,** Single amoeba images were further gated by side scatter (channel 6) and channel 2 intensity. **e,** The percentage of amoebae that were dead were then gated by side scatter and channel 7 intensities (Live/Dead violet).

**Supplemental Figure 2.** **Optimization of mouse serum supplementation with CaCl_2_ and MgCl_2_.** CMFDA-labeled amoebae were exposed to mouse serum for thirty minutes. Amoebae were stained with Live/Dead fixable violet and analyzed with imaging flow cytometry. To determine the appropriate concentration of CaCl_2_ and MgCl_2_ for mouse serum, amoebae were exposed to multiple different concentrations of both while also using human serum with normal supplementation as a positive control. The maximum amount of CaCl_2_ and MgCl_2_ to be used was based off of the work of Morrison et al (1990). Low corresponds to 15 mM CaCl_2_ and 50 mM MgCl_2_, medium corresponds to 45 mM CaCl_2_ and 150 mM MgCl_2_, and high corresponds to 75 mM CaCl_2_ and 250 mM MgCl_2_. N=2 across 1 experiment. Data were analyzed with Brown-Forsythe and Welch’s ANOVA tests using Dunnett’s T3 multiple comparisons tests with statistical significance indicated as follows: ns, *P* > .05; *, *P* ≤ 0.05; **, *P* ≤ 0.01; ***, *P* ≤ 0.001; ****, *P* ≤ 0.0001.

**Supplemental Figure 3.** **Immunofluorescence assays of human Jurkat T cells.** Human Jurkat T cells were labeled with CD3, CD46, or CD55 antibodies. Note the mean fluorescence intensity of CD3 staining is much higher than CD46 or CD55. Data are from a single experiment with n=1 for each antibody. Statistical analysis was not performed due to lack of replicates.

**Supplemental Figure 4. Non-normalized data from Figure 5b.** CMFDA-labeled amoebae were co-incubated with CMTPX-labeled Sf9 cells for 1 hour, or incubated in the absence of Sf9 cells, and then exposed to human serum for thirty minutes. Cells were stained with Live/Dead fixable violet and analyzed with imaging flow cytometry. N=12-15 across 5 independent experiments. Data were analyzed with Brown-Forsythe and Welch’s ANOVA tests using Dunnett’s T3 multiple comparisons tests with statistical significance indicated as follows: ns, *P* > .05; *, *P* ≤ 0.05; **, *P* ≤ 0.01; ***, *P* ≤ 0.001; ****, *P* ≤ 0.0001.

**SUPPLEMENTAL VIDEO LEGENDS**

**Supplemental Video 1. Live-cell imaging of amoebic trogocytosis of Sf9 cells, example 1.** CMFDA-labeled *E. histolytica* trophozoites (turquoise) were combined with CMTPX-labeled Sf9 cells (orange). The amoeba is already performing trogocytosis on the Sf9 cell, with a long narrow portion of the Sf9 cell visible within the amoeba, together with distinct bites of Sf9 cell material. The video first shows the merged imagery with both the amoeba and Sf9 cells visible, and then the video shows the Sf9 channel alone so that it is easier to see the orange Sf9 material within the amoeba. Images were collected using a Zeiss LSM 980 with Airyscan2 using multiple z-stacks from a top-down view for 3D imaging.

**Supplemental Video 2. Live-cell imaging of amoebic trogocytosis of Sf9 cells, example 2.** CMFDA-labeled *E. histolytica* trophozoites (turquoise) were combined with CMTPX-labeled Sf9 cells (orange). In this example, one amoeba is performing trogocytosis on at least two Sf9 cells simultaneously. The video shows the merged imagery with both the amoeba and Sf9 cells visible. Images were collected using a Zeiss LSM 980 with Airyscan2 using multiple z-stacks from a top-down view for 3D imaging.

**Supplemental Video 3. Live-cell imaging of amoebic trogocytosis of Sf9 cells, single channel view of example 2.** This video is identical to Supplemental Video 2, but here, only the Sf9 channel is shown, so that it is easier to see the orange Sf9 material within the amoeba.
